# Heparin-binding protein (HBP) worsens the severity of pancreatic necrosis via up-regulated M1 macrophages activation in acute pancreatitis mouse models

**DOI:** 10.1080/21655979.2021.2011018

**Published:** 2021-12-11

**Authors:** Liangliang Zhou, Jianjun Chen, Genhua Mu, Zhongqian Lu, Weiqin Li, Yijun Deng

**Affiliations:** Department of Intensive Care Medicine, Yancheng First Hospital, Affiliated Hospital of Nanjing University Medical School/The First People’s Hospital of Yancheng, Yancheng, Jiangsu Province, China

**Keywords:** Acute pancreatitis, m1 macrophages, heparin-binding protein

## Abstract

Acute pancreatitis (AP) is one of the most widespread clinical emergencies. Macrophages are the most common immune cells in AP pancreatic tissue and are closely associated with pancreatic necrosis and recovery. The level of heparin-binding protein (HBP) is closely linked to inflammation. In this study, we assessed the effect of HBP on AP tissue necrosis severity and whether HBP is associated with M1 macrophages in pancreatic necrosis. We observed the dynamic changes of HBP levels in the pancreas during acute inflammation in the caerulein-induced AP mice model. We used hematoxylin–eosin staining to evaluate pancreatic edema and necrosis, and to detect infiltration of macrophages by immunohistochemistry. Moreover, expressions of the maker and cytokines of macrophages, including inducible nitric oxide synthase (iNOS), and arginase 1 (Arg-1), interleukin-1β (IL-1β), tumor necrosis factor-α (TNF-α), and interleukin-6 (IL-6) mRNA, were detected by real-time polymerase-chain reaction (RT-PCR). High levels of HBP in the pancreas were detected at 48 h, and heparin inhibited HBP expression in AP pancreatic tissue. Inhibiting HBP expression by injecting heparin before AP can alleviate pancreatic necrosis and inhibit F4/80 labeled M1 macrophage infiltration and IL-6, TNF-α, and iNOS mRNA expression. Clodronate liposome (CLDL) intraperitoneally treated mice showed no change in pancreatic HBP levels, but pancreatic macrophage-specific antigen F4/80 and TNF-α, IL-1β, and IL-6 mRNA levels decreased after CLDL treatment. HBP is critical for pancreatic necrosis response in acute pancreatitis by increasing the infiltration of M1 macrophages and promoting the secretion of inflammatory factors, such as TNF-α, IL-6, IL-1β, which can be reduced by heparin.

## Introduction

Acute pancreatitis (AP) is a frequent digestive disease with high morbidity and mortality [1–3]. In recent years, patients with severe pancreatitis have still died from pancreatic necrosis, infection, systemic organ failure, and other complications [1,4–6]. Importantly, there are currently no treatments that can change the disease course [7]. Early prediction of disease severity is important for pancreatitis and can improve patient prognosis [8]. However, currently, commonly used indicators are not specific enough to reflect the seriousness of the disease [9], so it is necessary to find new indicators for evaluation. Multiple pathophysiological factors contribute to acute pancreatitis, including inflammatory storms, tissue inflammation, vascular leakage, and cell necrosis. At present, the pathogenesis of AP has been demonstrated to be caused by pancreatic digestive enzymes, inflammatory cell infiltration, pancreatic tissue leakage, and pancreatic necrosis [5,10,11]. Intravenous infusion, anticoagulation, analgesia, and elimination of inflammatory factors are essential for AP treatment [1]. Among these elements, the critical mediator seems to be pancreatic tissue inflammation. The latest research has shown that innate immune and inflammatory signaling pathways participate in acute pancreatitis pathogenesis. These processes are initiated by pancreatic immune cell infiltration and play an important role in pancreatic tissue injury.

Macrophages are the most customary immune cells inculpated in pancreatic injury and are closely related to pancreatic necrosis and recovery [12–14]. Due to different immune microenvironments, macrophages can be divided into various phenotypic and functional subtypes, showing assorted functions. In the initial acute pancreatitis, macrophages tend to differentiate into M1, producing inflammatory mediators and tissue necrosis [15]. M1 macrophages produce pro-inflammatory mediators, involving inducible nitric oxide synthase (iNOS) and tumor necrosis factor-α (TNF-α), enhancing reactive oxygen assembly. F4/80 is the special mark of M1. On the contrary, anti-inflammatory M2 macrophages up-regulate arginase-1 (Arg-1), scavenger, and mannose receptor, and are found in inflammatory zone 1 (FIZZ1) protein expressions. Thus, iNOS is widely employed as an M1 marker, and Arg-1 and FIZZ1 are classic M2 macrophage markers.

Low-molecular-weight heparin has an antithrombin activity and inhibits the inflammatory cascade response by reducing the release of cytokines and inflammatory mediators [16]. Studies have shown that preprocedural heparin injection significantly reduces endoscopic retrograde cholangiopancreatography (ERCP)-associated pancreatitis [17]. Li et al. [18] demonstrated that prophylactic low-molecular heparin administration prevented severe acute pancreatitis in rats partially through the VEGF/Flt-1 signaling pathway. In recent years, several studies have confirmed that heparin-binding protein (HBP) levels were elevated in the plasma and tissues of patients with sepsis [19–21]. It is widely used as a severe infection development and prognosis prediction biomarker and has recently been suggested as a participant in the pathophysiology of AP. Interestingly, HBP has been reported to be raised in AP patient serum, and plasma HBP may be associated with severity necrosis of AP [22]. However, the expression of HBP in the pancreatic tissue and whether HBP is associated with AP’s immune injury mechanism have not been reported [22,23]. For chemokines of monocytes, T cells, and neutrophils, HBP promotes monocyte release of cytokines, phagocytosis, and endothelial adhesion. Fisher et al. [24] findings suggest that HBP causes inflammation and capillary leakage in multiple organs. However, whether HBP induces macrophages to stimulate pancreatic inflammation and necrosis in AP models is unknown. So far, the effect of HBP on AP tissue damage has never been assessed. Therefore, to investigate whether HPB can be used as a diagnostic indicator of AP and its association with macrophages, we established caerulein-induced AP mice models to detect HBP and M1-type macrophage-related expression factors. We then used clodronate liposome (CLDL) to deplete macrophages [25] as a reversion experiment to confirm the key role of macrophages in the process of HBP regulating the inflammatory response of AP and to provide new acute pancreatic cancer diagnostics and therapeutic strategies.

## Materials and methods

### Ethics statement

Wild-type (WT) mice (C57BL/6; 25–30 g) 6-to 8-weeks in age, matched by sex, are kept in the special pathogen-free cages with temperatures at 23 ± 2 °C and humidity of 60 ± 10%, a 12-h dark cycle of light/12 h. Unrestricted food and water access were provided. The Animal Care and Use Committee of the Yancheng First Hospital, Affiliated Hospital of Nanjing University Medical School has given approval to all procedures with the Guidelines for Animal Care and Use strictly followed.

### AP animal models

Caerulein was injected into the mice for acute pancreatitis induction [5]. Briefly, saline as control or 100 μg/kg caerulein (Sigma-Aldrich, USA) was intraperitoneally injected into mice (i.p., ten times hourly) with harvesting completed at designated time points. Mice (n = 6 for each group) were harvested at 0 h, 24 h, 48 h, 72 h, 120 h, and 144 h after induction of AP.

An intraperitoneal bolus of 0.4 U/g bodyweight of unfractionated heparin (AP+Hep group) or phosphate-buffered saline (AP+Placebo group) (Formumax, USA) was given to mice 12 h before caerulein-induced AP.

To study the relationship between HBP and M1 macrophages, 200 μL clodronate liposome (AP+CLDL group) or control liposome (AP+Lip group) (FormuMax Scientific, USA) was given to mice intraperitoneally once daily.

### Pancreatic histology analysis

CO_2_ inhalation was used to sacrifice mice, and the pancreas tissues were taken for histological observation and immunohistochemical examination. The pancreatic sections were immobilized with 10% formalin, embedded in paraffin, and used hematoxylin (H&E) to stain. The histological score was assessed according to previous reports. In short, 3–5 random images of each mouse pancreatic slide were assessed. The pancreatic necrosis (%) percentage was compared. Two pathologists evaluated the histological analysis using a blinded method to prevent bias; the severity of AP was compared with the placebo implanted group.

### Quantitative RT-PCR (qRT-PCR)

The pancreatic tissue is lyzed using the Trizol reagent (Invitrogen, USA), and the total tissue RNA is extracted. A high-capacity cDNA reverse transcription kit (using a biological system) was utilized to reverse transcribe total RNA (5 mg) into cDNA and detect mRNA expression of these genes using real-time PCR using the ABI Step One-Step system (Fast Start Universal SYBR Green Master; Roche, Shanghai, China). The 2^−ΔΔCT^ method was used to analyze the results gained with all gene expression levels normalized to β-actin. Primer sequences are shown in Table 1.

**Table 1.** Primers for qRT-PCR

Gene		Primer sequences
FIZZ-1	Forward	5ʹ-TGGCTTGCGAGACGTAGAC-3’
	Reverse	5ʹ-GCTCAGGTGAATCGGCCTTTT-3’
Arg1	Forward	5ʹ-CTCCAAGCCAAAGTCCTTAGAG-3’
	Reverse	5ʹ-AGGAGCTGTCATTAGGGACATC-3ʹ
iNOS	Forward	5ʹ-AGGGAATCTTGGAGCGAGTT-3ʹ
	Reverse	5ʹ-GCAGCCTCTTGTCTTTGACC-3ʹ
TNF-α	Forward	5ʹ-TCTCTTCAAGGGACAAGGCTG-3ʹ
	Reverse	5ʹ-ATAGCAAATCGGCTGACGGT-3ʹ
IL-1β	Forward	5ʹ-TTGACGGACCCCAAAAGAT-3ʹ
	Reverse	5ʹ-TTCATTCTCTTTGCTCTTGAATTAGA-3ʹ
IL-6	Forward	5ʹ-GAAGCTGGATGCTCTCATCTG-3ʹ
	Reverse	5ʹ-GTCTGACCTTTAGCTTCAAATCCT-3ʹ
β-actin	Forward	5ʹ-CAGTAACAGTCCGCCTAGAA-3ʹ
	Reverse	5ʹ-GATTACTGCTCTGGCTCCTA-3’

### Immunohistochemistry

Pancreatic tissue sections were stained with F4/80 immunochemistry. Pancreas slices of 3 mm thickness were dewaxed and rehydrated with serial alcohol. Endogenous peroxidase activity was inhibited by soaking the slices in 3% hydrogen peroxide for 10 min. Incubation was then done with buffered normal horse serum to inhibit nonspecific binding. Prior to immunohistochemistry, the specimens were immersed in 0.1 mol/L citrate buffer (pH 6.0) for 25 min and then heated in a voltage cooker for 5 mins for antigen repair. Slices were incubated with mice against F4/80 monoclonal antibodies (1: 100, Abcam, United States) at 4°C. Goat antirabbit or rat antirat-resistant secondary antibodies (ShanghaiLongisland Biotechnology Co., Ltd., Shanghai) coupled with horseradish peroxidase (HRP) were incubated at room temperature for 1 hour. Next, 3,3-diaminobenzidine tetrahydrochloride (DAB, ShanghaiLongisland Biotechnology Co., Ltd., Shanghai) was coated brown. Finally, the specimens were restained with hematoxylin, and an Olympus camera took microscopic photos.

### Western blot

Whole pancreatic tissue extracts were set up. The protein sample was dissolved with 10% SDS-PAGE gel, transported to the PVDF membrane before sealing with 5% skim milk. Afterward, overnight incubation at 4°C with the primary antibody against HBP (1:1000; Abcam, United States) was done, then coupled with horseradish peroxidase for 1.5 h to detect the protein bands with an enhanced chemiluminescence (ECL) detection system (AGCLS, Rockford).

### Statistical analysis

Mean ± standard deviation (SD) was used to express the gained data. Following the assessment of the normally distributed data with the application of the Kolmogorov–Smirnov test, the Student’s *t*-test was utilized to evaluate statistical comparison among the experimental groups. Significance was considered with *P* < 0.05 on the one-way ANOVA with Prism 6 GraphPad Software.

## Results

Whether HBP directly causes pathological damage to pancreatic tissue or indirectly regulates the inflammatory response of the pancreas by modulating the phenotype of macrophages is important. Our findings support the second hypothesis. We designed a response experiment and found that when chlorophosphate liposomes were injected tail vein to remove macrophages from mice, HBP remained elevated in the animal model of pancreatitis, but the pancreatic damage caused by the elevated HBP was significantly reduced; whereas when only liposome carriers were used without macrophage removal, HBP expression levels remained similarly elevated, while HBP damage to pancreatic tissue was not different from that in the control model of pancreatitis. There was no difference compared to the control model of pancreatitis. Therefore, we believe our hypothesis that HBP exacerbates pancreatic injury in the mouse model of AP through M1-type macrophage activation.

### HBP levels and M1-type macrophages were increased in caerulein-induced AP

**Figure 1.**
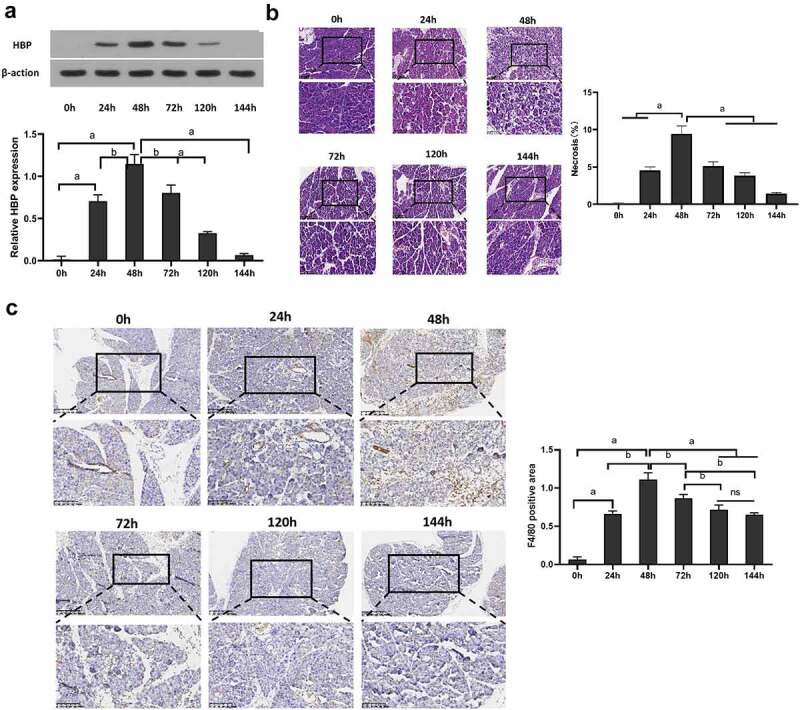
HBP expression level in pancreatic tissue and level of M1 macrophage following AP injury. (a) Western blot and qRT-PCR were used to detect the relative expression level of HBP protein and mRNA in pancreatic tissue; (b) Pancreatic pathological change at different times assessed using H&E staining; (c) Immunohistochemistry assess the level of F4/80 in Pancreatic tissue. Data are Means ± SD, n = 6 for each group, ^a^*P* < 0.01, ^b^*P* < 0.05

For detecting caerulein-induced AP pancreatic tissue HBP production, mice were given intraperitoneal injection and euthanasia at different times. The result showed that the level of HBP protein and mRNA in pancreatic tissue was low in saline-injected mice. However, in the AP mice model, pancreatic HBP production increased significantly within 0–48 h, followed by a gradual decline over the next few days Figure 1(a). Histological staining to assess tissue necrosis found that pancreatic edema increased significantly, marked areas of focal necrosis and destruction of lobular structures after induction of AP compared to saline-injected mice, and tissue necrosis reaching its maximum extent within 48 h Figure 1(b). Immunohistochemistry showed that macrophages rise rapidly at 48 h and remain at high levels Figure 1(c).

### Reducing HBP reduced pancreatic tissue necrosis and M1 macrophage level

**Figure 2.**
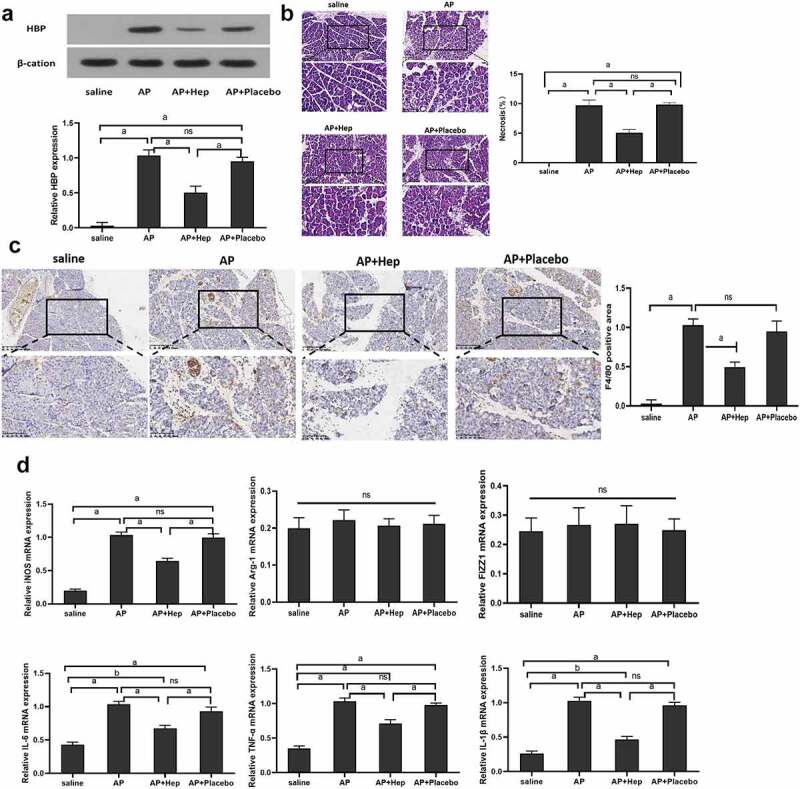
The effect of reducing HBP on pancreatic tissue necrosis and the level of M1 macrophages. (a) The level of HBP expression in different groups at 48 h detected by Western blot and qRT-PCR; (b) 48 h pancreatic pathological change assessed via H&E staining. Magnify it 200 times; (c) Immunohistochemistry assess the level of F4/80 in Pancreatic tissue at 48 h; (d) qRT-PCR detected the levels of M1 and M2 macrophage-related markers. Magnify it 200 times. Data are Means ± SD, n = 6 for each group. ^a^*P* < 0.01, ^b^*P* < 0.05

Pancreatic tissue HBP protein and mRNA expression were significantly inhibited by heparin in the AP model Figure 2(a). A biopsy of the pancreas was histologically examined 48 h after the induction of AP. The AP model observed the pancreas’ tissue damage, which manifested as immune cells’ infiltration, tissue swelling, exudation, and pancreatic necrosis. The rate of pancreatic tissue necrosis was significantly reduced after reducing HBP Figure 2(b). An antibody against mouse macrophage F4/80 was applied to stain pancreatic tissue sections for assessing the extent of macrophage infiltration prior to AP. M1 specific antigen F4/80 expression in the AP+heparin group was lower when in comparison with the AP and AP+Placebo groups Figure 2(c). Furthermore, it was discovered that iNOS, TNF-α, IL-1β and IL-6 expression, which is the marker of M1 type, in the AP +heparin group, was lesser when in comparison with the AP group and AP+Placebo. There was no significant difference seen in the Arg-1 and FIZZ1 expression in different groups Figure 2(d).

### HBP aggravates AP pancreatic necrosis dependent on M1 macrophage

**Figure 3.**
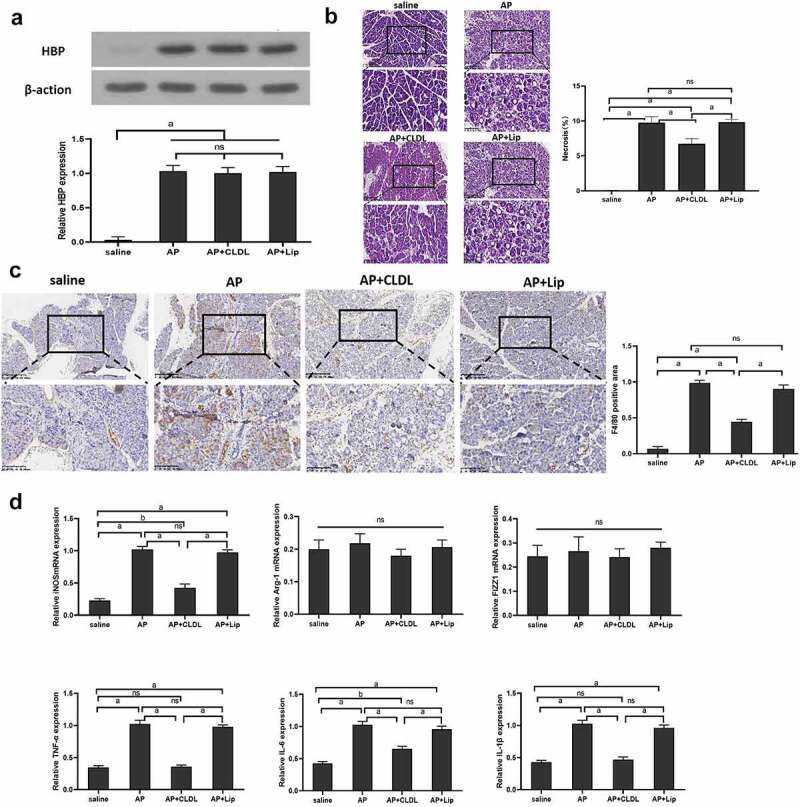
Specific roles of M1 macrophage in the early stages of AP injury

To further study the relationship between HBP and M1 macrophages, pancreatic macrophages are depleted on Day 1 and Day 2 following caerulein-induced AP by Clodronate liposome (AP+CLDL) or control liposome (AP+Lip) intraperitoneal injection. We observed pancreatic tissue HBP expression in mice at 48 h after administration of AP, AP+CLDL, and AP+Lip, but the difference was not significant between the three groups Figure 3(a). The level of pancreatic tissue necrosis and the expression of M1 macrophage marker F4/80 in the AP+CLDL group were lesser when in comparison with the AP group and AP+Lip group, and the expression of iNOS, TNF-α, IL-6, and IL-1β in the AP+CLDL group was lower than that in the AP group and AP+Lip. However, expression of FIZZ1 and Arg-1mRNA was low in all samples, and no considerable discrepancy was observed among the groups Figure 3(b-d), indicating that HBP aggravated pancreatic necrosis by macrophage M1 activation.

(A) The level of HBP expression in different groups at 48 h detected by Western blot and qRT-PCR; (B) 48 h pancreatic pathological changes in other groups assessed via H&E staining. Magnify it 200 times; (C) immunohistochemistry assessing the level of F4/80 in Pancreatic tissue in different group at 48 h; (D) qRT-PCR detected the levels of M1 and M2 macrophages related markers in other groups. Magnify it 200 times. Data are means ± SD, n = 6 for each group; ^a^*P* < 0.01, ^b^*P* < 0.05.

## Discussion

Acute pancreatitis (AP) is a commonly known digestive system inflammatory disease coupled with prominent morbidity and mortality [1]. The degree of pancreatic necrosis is closely related to morbidity and mortality [26]. Yet, the pathogenesis of acute pancreatitis remains debatable [27]. Therefore, it is essential to understand pancreatic necrosis’s molecular and cellular mechanisms in AP, and it is necessary to diagnose and treat pancreatic necrosis effectively. Multifunctional cells from the innate immune system known as macrophages are critical in AP necrosis development, be considered essential mediators of acute pancreatic necrosis in animal models [28,29]. The inflammation reaction is in intimate association with macrophage activation [28,30]. Macrophages are mainly divided into M1 and M2 types; M1 demonstrates strong pro-inflammatory activity, whereas M2 participates in inflammation mitigation and tissue repair [6,10,12]. M1 macrophages play a crucial role in antigen presentation, pro-inflammatory cytokine secretion, and phagocytic activity based on the potential mechanism [12,13]. In the present study, we found that M1 macrophage levels were significantly increased in the pancreas of caerulein-induced AP mice, and levels of inflammatory factors TNF-α, IL-6, and IL-1β were significantly elevated. Han et al. [31] showed that myeloid-specific dopamine D2 receptor signaling can control the inflammatory response to AP by inhibiting M1 macrophages. This suggests that inhibition of the pro-inflammatory activity of M1 macrophages could reduce AP necrosis.

Studies have shown that severe pancreatitis patients had higher HBP levels than patients and animals with pancreatitis [22,32]. HBP increases pancreatic necrosis and is vital in pancreatic injury-associated initial inflammatory response [10,22,32]. HBP has been shown to interact with various immune cells and play a role in multiple organ failure [20,33]. Recent proof from experimental research has indicated that heparin suppressed HBP activation [33]. Heparin blocks HBP-related pro-inflammatory cytokine production, demonstrating a credible mechanism where heparin hinders ship likely by obstructing the HBP heparin-binding domain [33]. It was not clear whether elevated HBP levels merely reflected the severity of the injury or a causal relationship between HBP and pancreatitis. Our study showed that the expression of HBP in acute pancreatitis tissue was significantly up-regulated. Heparin treatment alleviated pancreatic necrosis after 48 h in cerulein-induced AP. Interestingly, macrophage infiltration was significantly reduced after heparin treatment, and the expression of inflammatory factors TNF-α, IL-6, and IL-1β was significantly decreased. This result is similar to the findings of Chen et al [34]. They found that Tetramethylpyrazine reduced the severity of AP by decreasing the levels of pro-inflammatory factors TNF-α and IL-6. According to these findings, HBP may promote AP pancreatic necrosis by activating the pro-inflammatory mechanism of M1 macrophages, while heparin inhibits this effect.

To further demonstrate that elevated HBP promotes AP pancreatic necrosis depending on M1 macrophage infiltration, we used the macrophage scavenger CLDL to inject the mice for scavenging macrophages *in vivo* [35]. We found no significant change in the HBP level in pancreatic tissue, but the macrophage-specific antigen F4/80 was immensely reduced. Pancreatic TNF-α and IL-6 levels also notably dropped following the treatment with the CLDL. These results suggest that M1 macrophages play a key role in the regulation of the inflammatory response to AP by HBP.

## Conclusions

In summary, HBP is vital in the initial inflammatory response and cell necrosis in AP by activating M1 macrophages and promoting TNF-α and IL-6 secretions. Therefore, the strategy of limiting the infiltration or activation of early macrophages may be a new method to prevent or treat AP, and the level of HBP may be used as an early diagnostic marker for AP. However, the molecular mechanism implicated in M1 macrophage activation needs to be studied in depth. Hence, this study helps improve understanding of the complex events concerned with early pancreatic necrosis in AP, which provides hope for early clinical usage of heparin as an effective treatment strategy.

## Data Availability

The data used to support the findings of this study are included within the article. Further inquiries can bedirected to the corresponding author, yysyy_icu@163.com

## Highlights

1. Heparin binding protein (HBP) levels and M1-type macrophages were increased in acute pancreatitis;
2. Reducing HBP can reduce pancreatic tissue necrosis and M1 macrophage level;
3. HBP aggravates AP pancreatic necrosis dependent on M1 macrophage.
